# The profile of orthopaedic sports medicine publishing in South Africa

**DOI:** 10.17159/2078-516X/2022/v34i1a14413

**Published:** 2022-01-01

**Authors:** BR Gelbart, E Schapkaitz, D Levitt

**Affiliations:** 1 Department of Orthopaedic Surgery and Wits Sport and Research group (WiSH), Faculty of Health Sciences, University of Witwatersrand Medical School, Johannesburg, South Africa; 2 Department of Molecular Medicine and Haematology, Faculty of Health Sciences, University of Witwatersrand Medical School, Johannesburg, South Africa; 3 Pre-Med Yeshiva College, Yeshiva University, New York, USA

**Keywords:** sports injury, knee injury, shoulder injury, rugby, soccer, levels of evidence, sports research

## Abstract

**Background:**

The South African Journal of Sports Medicine (SAJSM) and the South African Orthopaedic Journal (SAOJ) are two open access, peer-reviewed journals which provide ongoing education to the sports medicine community.

**Objectives:**

The purpose of this review was to appraise articles with a sports orthopaedic focus published in SAJSM and SAOJ. A secondary aim was to evaluate trends regarding the focus of the articles, levels of evidence, authors’ affiliations, and country of origin.

**Methods:**

An electronic search of the SAJSM from 1982 to 2021 and SAOJ from 2008 to 2021 was conducted to identify relevant articles. The eligibility of the articles was determined according to the following inclusion criteria: SAJSM articles with reference to musculoskeletal anatomy and/or an injury in any sport, and SAOJ articles focusing specifically on sports, sports teams and low-velocity traumatic injuries in sports people.

**Results:**

This study included specific sports orthopaedic articles in SAJSM (n=161) and SAOJ (n=41). The articles originated from 67 institutions in 19 countries. In SAJSM, the majority of articles were published by local authors (n=44, 61%). There was a non-significant difference in the proportion of articles from local and international institutions in SAOJ. In SAJSM, the sports covered most frequently included rugby, cricket, running and soccer, whereas in SAOJ most articles referred to low-velocity injuries. With regard to trend analysis, a significant decline in articles with Level V evidence published by SAJSM was observed (p<0.001). Similarly, articles with Level V evidence published by SAOJ showed a decline, although it was non-significant.

**Conclusion:**

The focus of SAJSM in particular is relevant to sports played, injury patterns and the healthcare resources for sports people in South Africa. The level of evidence published by SAJSM has improved significantly over time.

Recently, orthopaedic sports medicine has been recognised as a sub-speciality of orthopaedic surgery. For example, in the United States of America, many professional sports teams include orthopaedic surgeons among the team’s complement of treating physicians. In other countries, and to some extent in South Africa, the medical care of professional sports teams is primarily managed by general practitioners (GPs), who also have qualifications in sports medicine. This further sub-specialisation is necessary in order to best manage the sports person’s health and performance, in addition to injuries of the skeletal system. Specialist orthopaedic care consultations are often required in particular for cases outside of the qualifications and capabilities of the team doctors. Orthopaedic surgeons may specialise in a particular joint or area of orthopaedics, including trauma. It is therefore essential to recruit orthopaedic specialists who understand the pressure and demands placed on the professional sportsperson. Furthermore, there are many patients who present to GPs or specialists who are amateur or recreational sportspeople. For these individuals, returning to sports after injury is also important. It takes clinical skill, knowledge and experience to customise the treatment to the needs of the level of the athlete.

South Africa’s climate and infrastructure allows for a wide range of sports participation. Exercise has been pioneered as a complementary approach to modifying lives and lifestyles. ^[1, 2]^ However, the increase in unfit and underprepared people who may be chasing the benefits of sports participation places a significant demand on GPs and specialists.

The South African Journal of Sports Medicine (SAJSM) and the South African Orthopaedic Journal (SAOJ) are open access, peer-reviewed journals that have provided ongoing education to the sports medicine community. SAJSM was launched in 1982 with a significant representation of orthopaedic surgeons on the Editorial Board. The journal’s scope has evolved to include sports medicine, biokinetics, physiotherapy, exercise and sports science, dietetics, and psychology, with particular relevance to South Africa. ^[3]^ In 2002, SAOJ was founded as the official publication of the South African Orthopaedic Association. It focuses specifically on orthopaedic surgery in South Africa, with sub-disciplines of relevance to orthopaedic surgeons. These include paediatrics, hip, knee, tumour and sepsis, spine, shoulder and elbow, foot and ankle and hand surgery. ^[4]^ There are a number of benefits to having an open access journal, with a specific emphasis on these aspects, and related to South Africa. In particular, the healthcare availability and resources for sportspeople differ according to country and region.

A narrative review was conducted to appraise the content of these two South African journals, with a particular focus on sports orthopaedics. The primary aim of the study was to assess the articles in each journal according to each anatomical region, as well as a relationship to sports. The secondary aims were to assess the trends regarding the focus of the articles, the levels of evidence, and the origins of the research.

## Methods

A search of the SAJSM and SAOJ was conducted electronically to identify online relevant articles using the table of contents. The SAJSM was accessed through the ‘Archives’ tab from 1982 to 2021. The year 2002, however, was missing online, and therefore no data could be collected for that year. The SAOJ was accessed through the Archives tab from 2008 to 2021 (Issue 3).

### Inclusion criteria

In SAJSM, articles with reference to musculoskeletal anatomy and/or an injury in any sport were included. In SAOJ, articles focused specifically on sports, sports teams and low-velocity traumatic injuries.

### Exclusion criteria

Congress abstracts, errata, editorials and opinions were excluded. Trauma-related articles specifically mentioning gunshot wounds, high-velocity trauma or post-traumatic sepsis were also excluded.

### Study selection

The index of each journal was scanned and the total number of articles published were identified. Articles were initially screened against the review’s inclusion and exclusion criteria by title to assess the eligibility. An abstract and article review was also performed when eligibility was unclear by means of the title alone. The eligibility assessment was performed independently by two reviewers. Articles were classified as ‘definite sports orthopaedic’ if the sport was mentioned and ‘possible sports orthopaedic’ if the sport was not mentioned but the article was possibly relevant to sports orthopaedics. ‘Definite sports orthopaedic’ articles were included. The level of evidence was captured if available. The level of evidence for SAJSM was not indicated in the articles. However, the level of evidence for SAOJ was indicated in the articles from the years 2018 until the year 2021. The articles were reviewed and assigned a level of evidence based on the Oxford Centre for Evidence Based Medicine (OECBM) levels of evidence. ^[5]^ Additionally, information pertaining to each article, including the specific anatomical region, sport, university or institute affiliations, and country of origin of the authors, were recorded.

### Statistical analysis

A custom Excel spreadsheet was developed to collect the data. Statistical analysis was performed using Statistica 13.2 software (Palo Alto, California, USA). Categorical data was presented as frequencies and percentages. Comparisons were performed using a chi-square test or two-tailed Fisher’s exact test where necessary. Subgroup analysis, with multiple pairwise comparisons, was subsequently performed applying the Bonferroni correction with a p-value <0.017 considered statistically significant. Agreement between each reviewer was assessed using Cohen’s kappa (κ) coefficient.

## Results

**Fig. 1:**
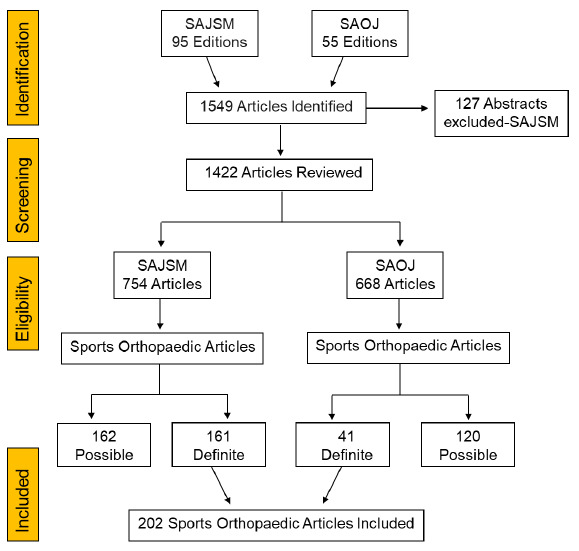
PRISMA flow diagram. SAJSM, the South African Journal of Sports Medicine; SAOJ, the South African Orthopaedic Journal

A search of 95 editions of SAJSM and 55 editions of SAOJ was conducted and the results are shown in Figure 1.

**Fig. 2:**
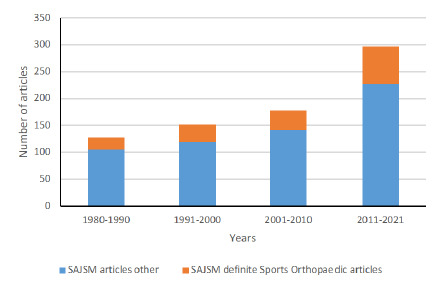
The trend of definite sports orthopaedic articles published in the South African Journal of Sports Medicine (SAJSM) between 1980 and 2021.

A total of 1549 articles were identified. Three SAJSM volumes which published 127 congress abstracts were excluded. Of the 1422 articles screened, there were 754 from SAJSM and 668 from SAOJ. In SAJSM, of the 162 (22%) eligible sports orthopaedic articles, 161 (21%) were definite. In SAOJ, of the 120 (18%) eligible sports orthopaedic articles, only 41 (6%) were definite. The interobserver agreement was Cohen’s kappa (κ) = 0.9 (95% CI, 0.8–0.9). As expected, the proportion of sports orthopaedic articles in SAOJ was lower than that in SAJSM (p<0.001). There was a non-significant increase in the number of definite sports orthopaedic articles published in SAJSM during the study period p=0.623 (Figure 2.).

**Fig. 3:**
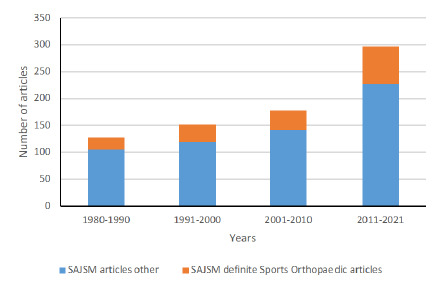
The trend of definite sports orthopaedic articles published in the South African Orthopaedic Journal (SAOJ) between 2008 and 2021.

In contrast, there was a non-significant decrease in the number of definite sports orthopaedic articles published in SAOJ during the study period (p=0.542) (Figure 3).

Articles were classified according to the anatomical focus (Table 1 and 2a and b) and also according to the sports concerned (Table 3). The most frequently reported anatomical regions in SAJSM were general anatomical sites (defined as more than one anatomical region), followed by specific anatomical regions namely; knee, shoulder and foot and ankle. The most frequently reported anatomical regions in SAOJ were specific anatomical regions, namely shoulders, c-spine, foot and ankle and knee.

On subgroup analysis between the four year groups in SAJSM, there was a significant decrease in articles with Level five evidence (p<0.001) (Table 4). There was, however, no significant difference for Levels one, two or three evidence between the four year groups. Level one to three evidence combined showed a non-significant increase between the four year groups (p=0.040). On subgroup analysis over the three year groups in SAOJ, there was no significant decrease in articles with Level five evidence (p=0.122). There was also no significant difference for Levels one, two or three evidence over the three year groups.

**Fig. 4:**
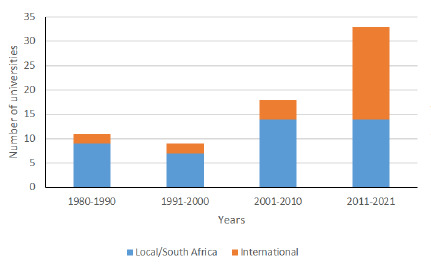
The proportion of local and international universities publishing in SAJSM

**Fig. 5:**
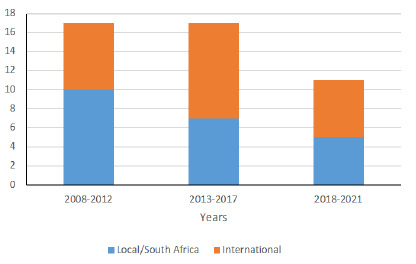
The proportion of local and international universities publishing in SAOJ

The articles originated from 67 universities and institutions from 61 cities in 19 countries (Supplementary Table 1.). In SAJSM, the majority of articles were published by local/South African authors (n=44, 61%). The proportion of articles from international universities in SAJSM increased during the four-year groups and approached statistical significance (p=0.059) (Figure 4). There was a non-significant difference in the proportion of articles from local and international universities in SAOJ between the three-year groups (p=0.569) (Figure 5).

## Discussion

The present study analysed recent trends in sports orthopaedic articles published in SAJSM and SAOJ. Similar studies have been previously performed. ^[6–8]^ These studies, however, applied narrower inclusion criteria and focused only on the level of evidence. Since 1980, 21% of articles published in SAJSM have been definite sports orthopaedic articles. The focus of the SAJSM is sports medicine and thus it is appropriate that of the 162 musculoskeletal articles published almost all were related to sports (99%). In contrast, 6% of the articles published in SAOJ since 2008 have been definite sports orthopaedic articles. This number increased to 18.0% when orthopaedic trauma articles relevant to sports orthopaedics were included.

The SAJSM has shown an increase in the number of articles published per decade since its inception. There has been a constant percentage of articles dedicated to sports orthopaedics, with no significant increase over time. In contrast, the more recent issues of SAOJ have shown a decrease in the absolute number of articles during its publication history. There has also been a non-significant decrease in definite sports orthopaedic articles that have been published in the SAOJ. This study did not investigate possible reasons for this, such as the journal’s editorial selection policy. Approximately half of the articles published in SAOJ were published by local/South African universities. In most of these academic institutions, sports orthopaedics is not practised as a dedicated sub-speciality. This limits the opportunity for training, as well as for orthopaedic surgeons to conduct sports orthopaedic research. This then may explain the lower number of sports orthopaedic articles in SAOJ.

A review of the sports focus published in SAJSM identified rugby, cricket, running and soccer as the most frequently published sports. The majority of articles, however, included more than one sports activity. In contrast, in SAOJ, most of the articles included were classified as ‘general’, which referred to the injuries that a sportsperson can sustain while playing sport. The second most common category was multiple sports, while only 4% of articles specified a particular sport. When one considers the frequent sports, in conjunction with the frequent anatomical regions, it appears that the focus has been on contact sports and those with a high physical demand. There may be scope for the SAOJ to publish more sports-specific orthopaedic topics. However, this may be limited by the number of injuries. ^[6]^

The low level of evidence in the articles published by SAJSM and SAOJ may raise concerns. Factors such as limited access to research funding and resources are possible contributors among local authors. Nonetheless, over the year groups, articles with Level V evidence published by SAJSM decreased significantly. In keeping with this trend, articles with Level V evidence published by SAOJ have also shown a decline, albeit non-significant. Orthopaedic journals with a higher impact factor are more likely to publish Level I or 2 articles. ^[9]^ There have been a number of authors who do acknowledge the place of Level 3 and 4 evidence and we, therefore, support the continued publishing of these studies. ^[6,10,11]^ However, authors should be encouraged to include control groups and to try and aim for a higher level of evidence. ^[10,11]^

Both journals showed a wide geographical and academic base of authors. The majority were published by the University of Cape Town. In SAJSM, articles were mainly published by authors from South Africa. This highlights the local relevance of the research published by SAJSM. It is interesting to note, however, that the proportion of articles from international universities increased over time. In contrast, articles in SAOJ were from both local and international universities.

The results of this review must be interpreted in the light of certain limitations. Firstly, while articles for SAJSM were available from 1980, articles for SAOJ were only available from 2008, which limits comparison. Secondly, this study investigated the level of evidence. In all articles for SAJSM and SAOJ (2008 to 2017), no level of evidence was available, and the articles were reviewed and assigned a level of evidence based on the OECBM. ^[5]^ However, these articles were discussed and reviewed by two independent reviewers. Furthermore, previous studies have demonstrated acceptable interobserver agreement between epidemiology- and non-epidemiology-trained reviewers. ^[11,12]^ Lastly, this review was limited to SAJSM and SAOJ only and we did not search the international literature to assess the number of South African sports orthopaedics articles which were published in international journals.

## Conclusion

This narrative review analysed the publishing trends for sports orthopaedics in two relevant South African journals, namely, SAJSM and SAOJ. We describe a wide range of data including anatomical regions, sports, level of evidence and origin of the authors, which highlights areas of strength and weakness. It was promising to note a decrease in the proportion of Level V evidence. The focus, in particular of SAJSM, is relevant to South Africa’s popular sports and injury patterns. The majority of articles published in SAJSM were from local authors, which highlights the importance of publishing research specific to South Africa, the relevant sports played in our country, and the healthcare resources for sports people.

## Supplementary Information



## Figures and Tables

**Table 1:** Frequency of sports orthopaedic articles published in SAJSM and SAOJ according to the anatomical focus

Years	SAJSM Total	SAJSM Sports orthopaedic	SAJSM Specific anatomical region	SAJSM General	SAOJ Total	SAOJ Sports orthopaedic	SAOJ Specific anatomical region	SAOJ General
1980–1990	128	23 (18)	17 (13)	6 (5)				
1991–2000	151	32 (21)	23 (15)	9 (6)				
2001–2010	178	37 (20)	18 (10)	19 (11)	166	31 (19)	29 (18)	2 (1)
2011–2021	297	70 (24)	37 (13)	33 (11)	502	89 (18)	87 (17)	2 (0.4)

Data are expressed as n or n (%). General was defined as more than one anatomical region. Data for SAOJ was only available from 2008. SAJSM, the South African Journal of Sports Medicine; SAOJ, the South African Orthopaedic Journal.

**Table 2:** Anatomical focus of sports orthopaedic articles published in SAJSM and SAOJ

Anatomical region	SAJSM	SAOJ	Total
C-spine	7 (4)	14 (12)	21 (8)
T-spine	1 (0.6)	1 (1)	2 (1)
L-spine	7 (4)	3 (3)	10 (4)
Sacrum	2 (1)	2 (2)	4 (1)
Shoulder	16 (10)	28 (23)	44 (16)
Humerus	0 (0)	0 (0)	0 (0)
Elbow	2 (1)	4 (3)	6 (2)
Radius/Ulna	1 (0.6)	2 (2)	3 (1)
Wrist and hand	0 (0)	11 (9)	11 (4)
Pelvis	3 (2)	0 (0)	3 (1)
Hip	7 (4)	3 (3)	10 (4)
Femur	2 (1)	3 (3)	5 (2)
Knee	24 (15)	18 (15)	42 (15)
Tibia/fibula and compartments	5 (3)	7 (6)	12 (4)
Ankle and Foot	12 (7)	19 (16)	31 (11)
General	73 (45)	5 (4)	78 (28)

Data are expressed as n (%). General was defined as more than one anatomical region. SAJSM, the South African Journal of Sports Medicine; SAOJ, the South African Orthopaedic Journal.

**Table 3:** Frequency of sports orthopaedic articles published in SAJSM and SAOJ according to sport

Sport	SAJSM	SAOJ	Total
Running	15 (9)	1 (1)	16 (6)
Cricket	24 (15)	0 (0)	24 (9)
Rugby	34 (21)	0 (0)	34 (12)
Tennis	2 (1)	0 (0)	2 (1)
Dancing	1 (1)	0 (0)	1 (0.4)
Gymnastics	1 (1)	0 (0)	1 (0.4)
Aerobics	1 (1)	0 (0)	1 (0.4)
Soccer	11 (7)	0 (0)	11 (4)
Multiple sports	44 (27)	27 (23)	71 (25)
Military	2 (1)	1(0.8)	3 (1)
Baseball	1 (1)	0 (0)	1 (0.4)
Hockey	3 (2)	0 (0)	3 (1)
Cycling	4 (3)	1 (1)	5 (2)
Basketball	4 (3)	0 (0)	4 (1)
Olympics	2 (1)	0 (0)	2 (1)
Volleyball	1 (1)	0 (0)	1 (0.4)
Swimming	1 (1)	0 (0)	1 (0.4)
Squash	1 (1)	0 (0)	1 (0.4)
Golf	2 (1)	0 (0)	2 (1)
Karate	0 (0)	1 (1)	1 (0.4)
Paralympics	1 (1)	0 (0)	1 (0.4)
Rowing	2 (1)	0 (0)	2 (1)
Mixed Martial Arts	1 (1)	0 (0)	1 (0.4)
Ironman	1 (1)	0 (0)	1 (0.4)
Wheelchair Basketball	1 (1)	0 (0)	1 (0.4)
Netball	1 (1)	0 (0)	1 (0.4)
Ringball	1 (1)	0 (0)	1 (0.4)
Horse Riding	0 (0)	1 (1)	1 (0.4)
General Orthopaedics	0 (0)	88 (73)	88 (31)

Data are expressed as n (%). General Orthopaedics referred to injuries that a sportsperson can sustain while playing sport without mention of a specific sport. SAJSM, the South African Journal of Sports Medicine; SAOJ, the South African Orthopaedic Journal.

**Table 4:** Level of evidence of sports orthopaedic articles published in SAJSM and SAOJ

SAJSM						
Years	Total	Level 1	Level 2	Level 3	Level 4	Level 5
1980–1990	17	0 (0)	4 (24)	0 (0)	1 (6)	12 (70.6)
1991–2000	37	0 (0)	5 (14)	5 (14)	8 (22)	19 (51.4)
2001–2010	36	0 (0)	10 (28)	10 (28)	13 (36)	3 (8.3)
2011–2021	71	4 (6)	14 (20)	12 (17)	29 (41)	12 (16.9)

**SAOJ**						
**Years**	**Total**	**Level 1**	**Level 2**	**Level 3**	**Level 4**	**Level 5**

2008–2012	46	1 (2)	1 (2)	3 (7)	19 (41)	22 (48)
2013–2017	49	2 (4)	2 (4)	6 (12)	22 (45)	17 (35)
2018–2021	25	1 (4)	0 (0)	0 (0)	18 (72)	6 (24)

Data are expressed as n or n (%). Level 1 refers to systematic review of randomised trials; Level 2 refers to randomised trials; Level 3 refers to non-randomised trials/cohort studies; Level 4 refers to case-series, case-control, or historically controlled studies; Level 5 refers to expert opinion. SAJSM, the South African Journal of Sports Medicine; SAOJ, the South African Orthopaedic Journal.
