# Supplementary material for: The profile of orthopaedic sports medicine publishing in South Africa
Source: S Afr J Sports Med. 2022 Jan 1;34(1):v34i1a14413. doi: 10.17159/2078-516X/2022/v34i1a14413 (PMC9924608; doi:10.17159/2078-516X/2022/v34i1a14413)
Supplement: Supplementary file 1 [file 2078-516X-34-v34i1a14413-s001.pdf]

Table 1S. Origins of articles published in SAJSM and SAOJ

Institution, City, Country	SAJSM	SAOJ	Total
1= University of Cape Town	33	42	75
2 = Conradie hospital	2	0	2
3 = Military Institution	2	0	2
4 = University of Free State	5	3	8
5 = Cleveland clinic	1	0	1
6 = Children Hosp Massachusetts	1	0	1
7 = University of Witwatersrand	21	12	33
8 = University of Stellenbosch	21	14	35
9 = University of Fort Hare	2	0	2
10 = University of Zululand	6	0	6
11 = No University Affiliation	12	6	18
12 = University of Melbourne	2	1	3
13 = Sport Science Institute	5	0	5
14 = University of Pretoria	13	16	29
15 = University of Western Cape	2	0	2
16 = University of Natal (prior to name change)	1	0	1
17 =. University of Durban-Westville	2	0	2
18 = University of Port Elizabeth	1	0	1
19 = University of Johannesburg	5	0	5
20 = Nelson Mandela University	10	0	10
21 = University of Kwazulu-Natal (KZN)	12	13	25
22 = Macquarie University	1	0	1
23 = Centre for Sports Medicine and Orthopaedics	0	2	2
24 = Cape Town Shoulder Institute	0	6	6
25 = North West University	3	0	3
26 = Medical College Chandighar	1	0	1
27 = University of Sydney	1	0	1
28 = Uni versity of Utrecht	1	0	1
29 = University of Northern Iowa	1	0	1
30 = Leeds Beckett University	2	0	2
31 = Durban University of Technology	1	0	1
32 = University of West of England	1	0	1
33 = Jomo Kenyatta University	1	0	1
34 = University of Limpopo	0	3	3
35 = Sevem Deanery school of ICU	0	1	1
36 = Universal college of medical sciences	0	1	1
37 = Univ of medical science Shiraz	0	1	1
38 = Aga Khan University	0	2	2
39 = Medunsa	0	1	1

40 = Linkoping university	0	1	1
41 = Makerere University	0	1	1
42 = University of Amsterdam	2	2	4
43 = University of Minnesota	0	1	1
44 = Technikon Witwatersrand	2	0	2
45 = University of South Australia	2	0	2
46 = Barry University	1	0	1
47 = Catholic University of Leuven	6	0	6
48 = South African Medical research Council	2	0	2
49 = Griffith University	1	0	1
50 = Aspetar Orthopaedics and Sports Med	1	0	1
51 = International Olympic committee	5	0	5
52 = Vrije university Amsterdam	1	0	1
53 = Lunex university	1	0	1
54 = University of Bath	1	0	1
55 = University of Brighton	1	0	1
56 = Vu University Medical Centre	1	0	1
57 = University of New England NSW	1	0	1
58 = University of Ghent	0	1	1
59 = University of Shleswig Holstein	0	1	1
60 = University of Nairobi	0	0	0
61 = Government medical college Mewat	0	1	1
62 = South West peninsula school of graduate medicine	0	1	1
63 = John Radcliffe Hospital Oxford University	0	1	1
64 = University of Aberdeen	0	1	1
65 = University of Zimbabwe	0	1	1
66= Charite Universitatsmadedizia	0	1	1
67= Liverpool school of tropical medicine	0	1	1
